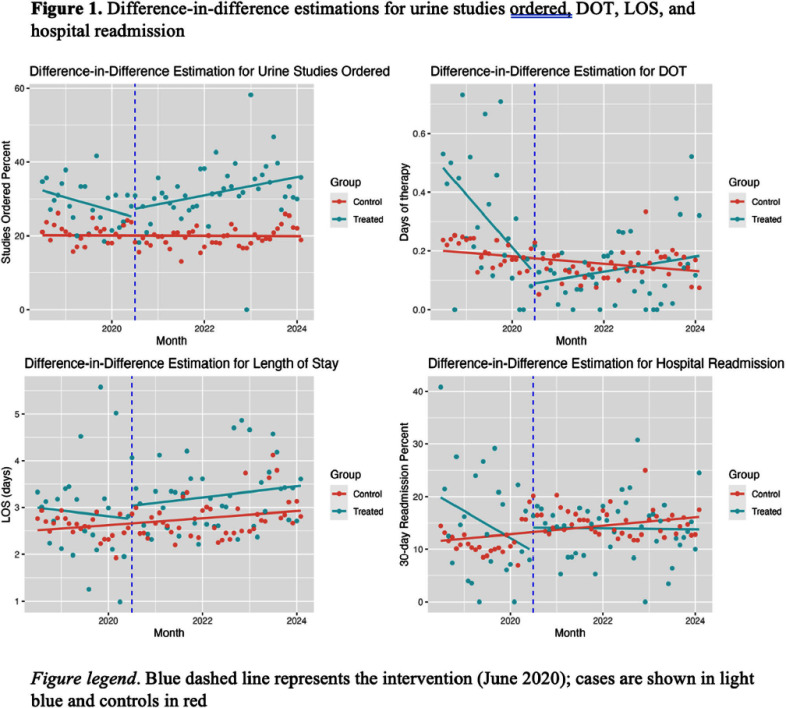# Assessing the Impact of an Electronic Health Record Intervention on Testing and Treatment for Asymptomatic Bacteriuria in Older Adults

**DOI:** 10.1017/ash.2025.390

**Published:** 2025-09-24

**Authors:** Eleanor Smith, Sabra Shay, Kady Phe, Andrei Zidaru, Mayar Al Mohajer

**Affiliations:** 1 Baylor College of Medicine; 2 Premier Inc; 3 Baylor St. Luke’s Medical Center; 4 BSLMC

## Abstract

**Background:** Asymptomatic bacteriuria (ASB) is common in older adults; however, its treatment in this population is not beneficial and can be harmful. We developed an intervention to update the indications on a urinalysis and urine culture order set according to recent Infectious Diseases Society of America (IDSA) guidelines recommending against testing and treatment for bacteriuria in older adults with altered mental status (AMS) or falls. **Methods:** This retrospective quasi-experimental study included adult patients > 65 years who presented to an Emergency Department (ED) in Southeast Texas from July 2018 to February 2024. The intervention, implemented in June 2020, included a change in the urinalysis (UA) with reflex to urine culture order set that replaced “AMS” as an indication for UA with “AMS and fever or leukocytosis” and provider education. The study’s primary outcome was whether urine studies were obtained within 24 hours of presentation to the ED. Secondary outcomes were antibiotic days of therapy (DOT) for suspected UTI, length of stay (LOS), and 30-day hospital readmission. The difference in differences (DID) technique was used to assess the impact of the intervention on older adults who presented with AMS or a fall (cases) compared to those with other chief complaints (controls). Outcomes were evaluated using multiple regression models (logistic for urinary studies ordered and readmission, linear for log LOS, and zero-inflated negative binomials for DOT) adjusted for patient demographics and comorbidities. **Results:** The study included 31,626 patients. Almost a third of older adult patients who presented to the ED with a fall or AMS (31.5%) received urinary testing, and 5.9% received antibiotics. 20.2% of control patients received urinary testing in the ED, and 6.3% received antibiotics. After adjusting for confounders, the intervention did not impact the percentage of tests ordered [(DID OR 1.14, 95% CI 0.93 – 1.40), (Figure 1)]. The incident rate ratio (IRR) of not receiving antibiotics was similar after the intervention (IRR 0.78, 95% CI 0.54 – 1.13), while the baseline number of antibiotics a patient received increased (IRR 1.54, 95% CI 1.05 – 2.27). **Conclusions:** Modifying UA indications on the urinalysis order set did not reduce testing or treatment for bacteriuria in older adults presenting with AMS or a fall. Longer-term or repetitive educational interventions could be an effective way to improve long-term stewardship outcomes in this population, especially for providers with less exposure to guidelines. Further discussion and investigation are needed.

**Figure:**